# Rectification of Priority: Giuseppe Vicentini (1860–1944) Presented the First Luminal Contrast Enhanced X‑Ray of the Digestive Tract on January 26, 1896

**DOI:** 10.5334/jbsr.3951

**Published:** 2025-04-25

**Authors:** Jean-François Monville, Robert F Dondelinger

**Affiliations:** 1 Department of Medical Imaging, Sankt Nikolaus Hospital, Eupen, Belgium

**Keywords:** Giuseppe Vicentini, rectification of priority, X‑rays, gastro‑intestinal tract, history of gastro‑intestinal radiology, 1896

## Abstract

Giuseppe Carlo Antonio Vicentini (1860–1944), professor of experimental physics at the University of Padova, Italy, presented on January 26, 1896, the X‑rays he made during a brief pioneering study period. Among those was the first roentgenograph of an opacified gastro‑intestinal tract, obtained of an animal model.

All publications on the history of radiology consulted by us take it for granted that Becher reported the first X‑ray of an opacified digestive tract on March 26, 1896.

In 1896, Dr Wolf Becher (1862–1906) was a medical practitioner in Berlin. He used a freshly sacrificed guinea pig for an experiment and gave a detailed description of his investigation [1]. After an abdominal midline incision, he isolated the gastric cavity, which was sutured at the cardia and the pylorus. Becher injected a solution of liquid lead acetate into the stomach by needle puncture. He had previously checked the high‑grade radio‑opacity of lead. During manipulation, the greater gastric curvature ruptured and was repaired by suturing. Then, Becher exteriorized an intestinal loop in the lower abdomen and filled it with the same solution. Some fur of the animal was contaminated by spoiled contrast. Becher carried the anatomical preparation to the workshop of the electro‑technician O. Messter, who operated an X‑ray facility a quarter of an hour from Becher’s workplace. During mobilization of the animal, the abdominal organs suffered from displacement and contrast escaped from the filled digestive cavities. An X‑ray was taken of the prepared guinea pig using a Hittorf vacuum tube and a Ruhmkorff induction coil, producing a spark of 15 cm. The distance from the tube to the object was 23 cm, and the exposure time was 35 min. The published X‑ray resulted in a print of inadequate quality due to the underexposure of the plate (Figure 1). The contrasted lumen of the exteriorized intestinal loop was visible, but the opacified stomach was not clearly recognizable. In a subsequently published paper devoted to gastric roentgenography after insufflation of air, the author added no more X‑rays, complaining that details had become lost due to the printing process [2]. Becher concluded that opacification of the gastro‑intestinal tract would be of interest for demonstrating fistulous tracts, provided the administered contrast exhibited intense opacity to the X‑rays and was harmless to the patient. Despite the nonprofessional laboratory production and the missing quality of the published roentgenograph, Becher is unanimously credited in the literature with the priority of having obtained the first X‑ray of the contrast‑enhanced lumen of the digestive tract. In his monumental biography of Wilhelm C. Röntgen and the history of the Röntgen rays, Otto Glasser refers to Becher as the initiator of roentgenographic visualization of the stomach with contrast material [3]. This assertion is incorrect.

**Figure 1:**
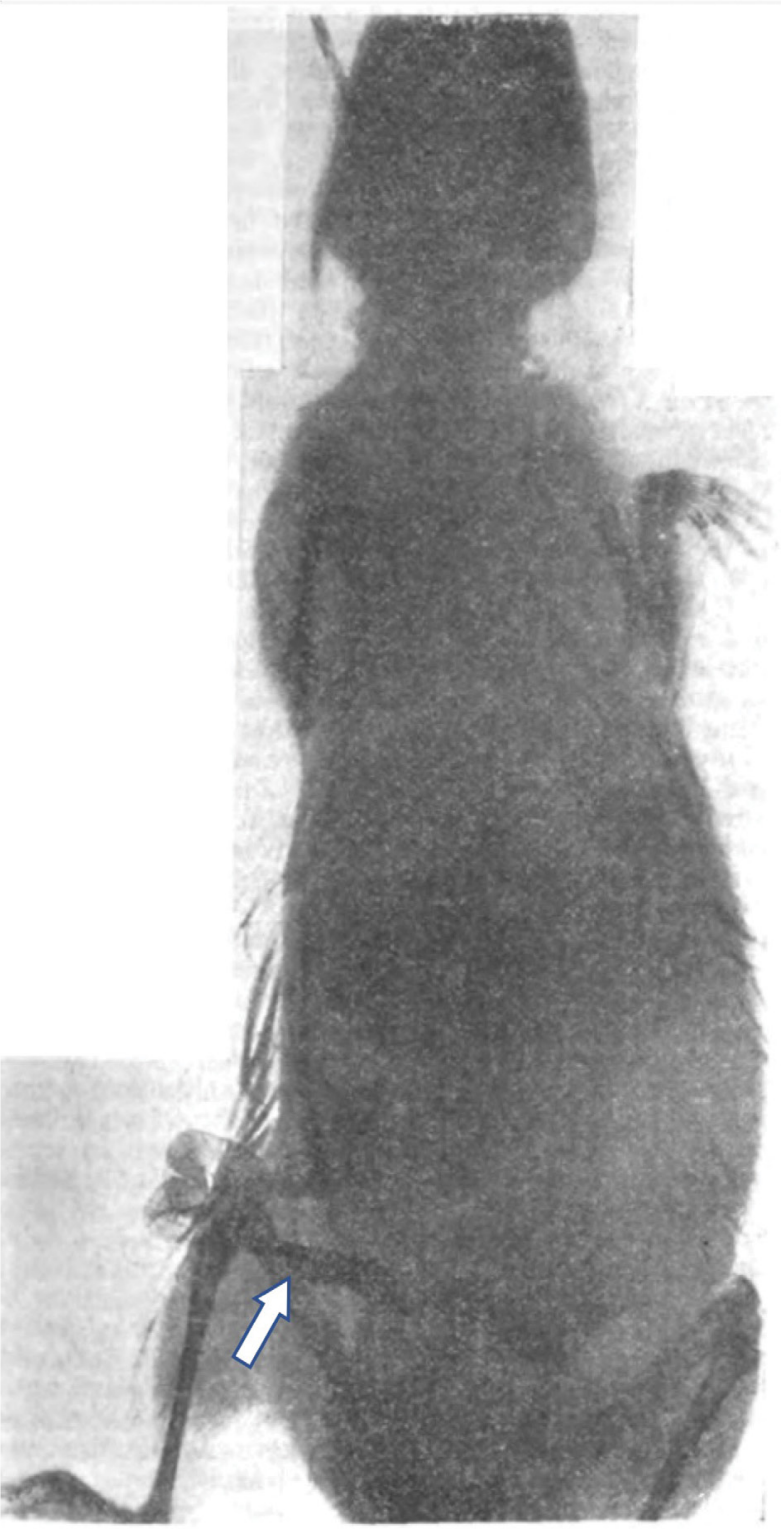
X‑ray of a guinea pig prepared by Becher. The exteriorized intestinal loop filled with a solution of lead acetate is shown (arrow). The stomach filled with contrast is barely visible.

Giuseppe Carlo Antonio Vicentini (1860–1944) (Figure 2) [4] was an extraordinary professor of experimental physics from 1894 to 1896, thereafter being an ordinary professor until 1931 and head of the Institute of Physics at the University of Padova, Italy, which in those days was installed at the Palazzo del Bo. Vicentini started experimenting soon after Röntgen’s discovery was announced in Italy by the *Corriere della Sera* in its issue of January 12–13, 1896 [5]. At the end of nine days of work, he produced, together with his first assistant Giulio Pacher (1867–1900), 13 roentgenographic plates, which showed diverse material including the skeleton of small animals and human hands and feet. Vicentini and Pacher were supported by Tulio Gnesotto (1871–1950), assistant at the Institute of Physics from 1894 until 1932. For the X‑rays taken in human, the physicists were advised by the surgeon Salvatore Catellani, an assistant to the professor of surgical pathology, Ernesto Tricomi (1859–1929). Vicentini employed a large Ruhmkorff coil connected to a Foucault‑like interrupter, accumulators, and a Crookes tube. The target to be radiographed was placed 10 cm from the vacuum tube. The exposure time was about 20 min for small objects. The quality of Vicentini’s X‑rays was such that one of them found its way into a popular American scientific magazine a few months later [6]. A large white mouse, with a body length of 20 cm and a belly of 4‑cm thickness was used for an experiment. Vicentini carried out no anatomical dissection of the animal but introduced a few drops of a mercury solution into the esophagus and a larger amount of the same contrast into the rectum. An X‑ray of the mouse was acquired in prone position, which depicted the contrast in the stomach, the rectum, and the cecum with reflux in the small intestine.

**Figure 2:**
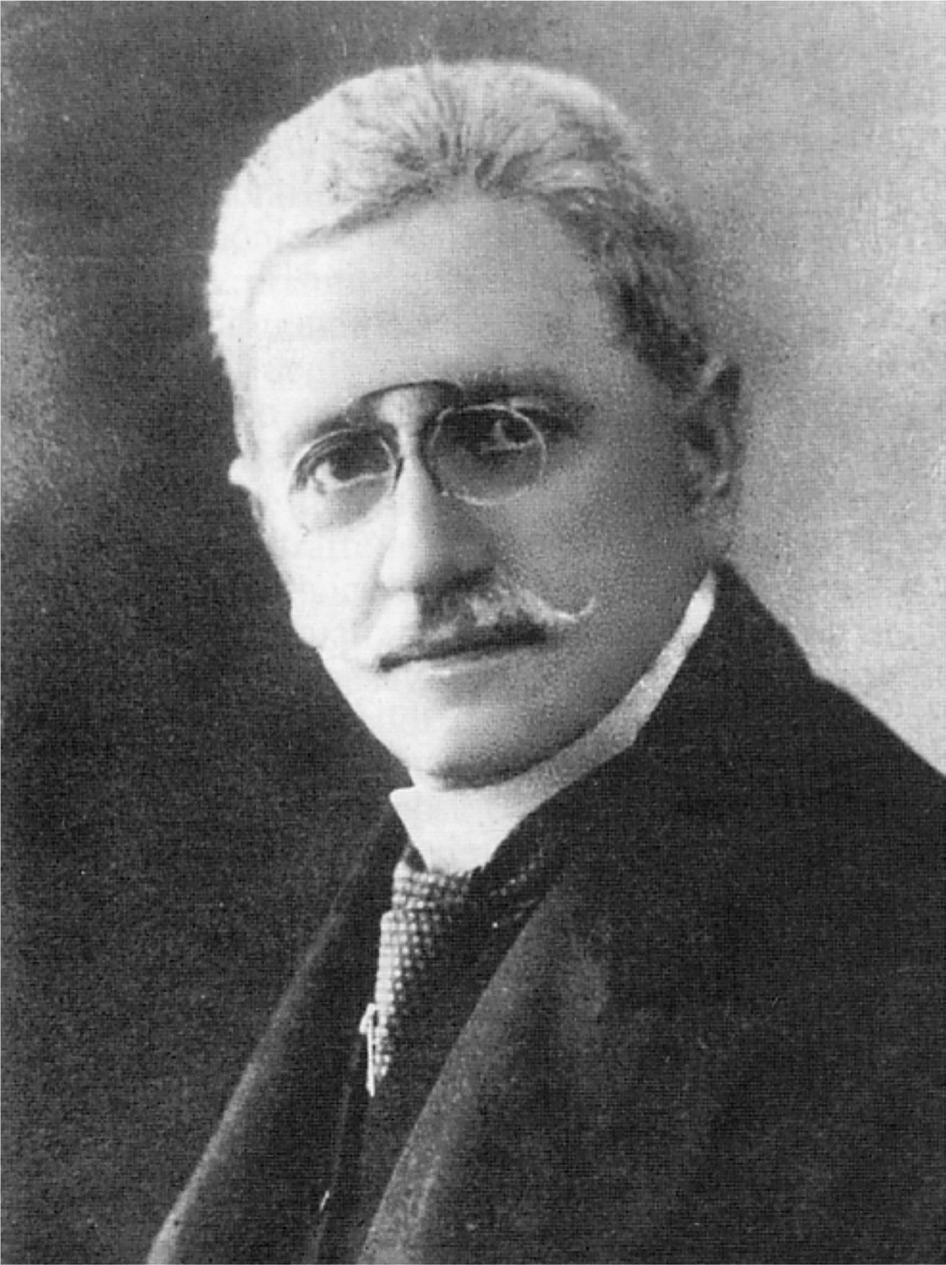
Portrait photograph of Professor Giuseppe Carlo Antonio Vicentini (1860–1944) [4].

The roentgenograph (Figure 3) was of demonstrative quality. Necropsy confirmed the X‑ray findings. At the request of the president in office, Fedele Lampertico (1833–1906), Vicentini presented on January 26, 1896, the result of his preliminary research to the Royal Institute Veneto of Sciences, Letters, and Arts, of which he was a member. An article, signed on January 25, was published in the *Memoirs of the Royal Institute*, comprising eight pictures at half actual scale, including the X‑ray of the mouse [7]. Vicentini formulated no comment on the potential diagnostic interest of contrast‑enhanced radiography of the gastro‑intestinal tract in medicine.

**Figure 3:**
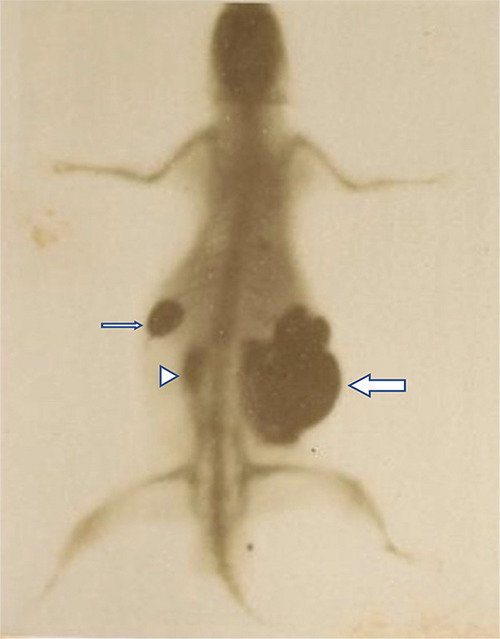
X‑ray of a white mouse set up by Vicentini: a solution of mercury introduced through the esophagus accumulated in the stomach (small arrow). The same solution infused through the anus is shown in the upper rectum (arrow head), the cecum, and intestinal loops (large arrow).

## Conclusion

Although it might sometimes seem hazardous to assert priority, there is inescapable evidence from the accessed published material that Vicentini’s animal experiment widely preceded the animal trial by Becher. Having uncovered no earlier reference in the literature, we suggest that Vicentini deserves, unless proven otherwise, the credit of priority for the first documented X‑ray of the opacified gastro‑intestinal tract.
